# New scoring system to identify RNA G-quadruplex folding

**DOI:** 10.1093/nar/gkt904

**Published:** 2013-10-08

**Authors:** Jean-Denis Beaudoin, Rachel Jodoin, Jean-Pierre Perreault

**Affiliations:** RNA Group/Groupe ARN, Département de biochimie, Faculté de médecine et des sciences de la santé, Pavillon de recherche appliquée au cancer, Université de Sherbrooke, QC J1E 4K8, Canada

## Abstract

G-quadruplexes (G4s) are non-canonical structures involved in many important cellular processes. To date, the prediction of potential G-quadruplex structures (PG4s) has been based almost exclusively on the sequence of interest agreeing with the algorithm G_x_-N-_1–7_-G_x_-N_1–7_-G_x_-N_1–7_-G_x_ (where x ≥ 3 and N = A, U, G or C). However, many sequences agreeing with this algorithm do not form G4s and are considered false-positive predictions. Here we show the RNA PG4 candidate in the 3′-untranslated region (UTR) of the TTYH1 gene to be one such false positive. Specifically, G4 folding was observed to be inhibited by the presence of multiple-cytosine tracks, located in the candidate’s genomic context, that adopted a Watson–Crick base-paired structure. Clearly, the neighbouring sequences of a PG4 may influence its folding. The secondary structure of 12 PG4 motifs along with either 15 or 50 nucleotides of their upstream and downstream genomic contexts were evaluated by in-line probing. Data permitted the development of a scoring system for the prediction of PG4s taking into account the effect of the neighbouring sequences. The accuracy of this scoring system was assessed by probing 14 other novel PG4 candidates retrieved in human 5′-UTRs. This new scoring system can be used, in combination with the standard algorithm, to better predict the folding of RNA G4s.

## INTRODUCTION

Guanine-rich nucleic acid sequences can fold into a non-canonical tetrahelical structure termed a G-quadruplex (G4). The primary building block of this structure, the G-tetrad, is composed of four coplanar guanines that interact with each other via Hoogsteen base pairs and are stabilized by a metal cation, usually potassium. The stacking of these G-tetrads forms a G4, which is a stable structure. Several bioinformatics studies reported enrichment in the number of potential G-quadruplex (PG4) sequences found in various DNA and RNA regulatory elements, respectively, located within the genome and the transcriptome (1–3). Promoters, telomeres and both the 5′- and 3′-untranslated regions of mRNA (UTRs) are some examples of these elements. Recently, an elegant approach based on an engineered structure-specific antibody led to the direct quantitative visualization of DNA G4s inside living cells (4). This study demonstrated that G4 formation in the nucleus of cells was modulated during cell-cycle progression, and that endogenous DNA G4s can be stabilized by a small-molecule ligand. Even though a quantitative, direct visualization of RNA G4 structures inside living cells is still lacking, over the past few years, several roles have been attributed to RNA G4s [for a review see (5)]. These include pre-mRNA splicing and polyadenylation, mRNA translation and targeting, transcriptional termination and telomere homeostasis (5). Clearly, RNA G4s appear to be one of the key motifs of the transcriptome. Thus, learning to accurately predict and locate RNA G4s is crucial to unlocking the study of their biological functions and impacts.

So far, most studies of biological G4 structures have combined bioinformatics predictions supported by physical evidence of G4 folding *in vitro*, as well as assessment of potential biological roles in cell culture assays [for examples see (6–8)]. A key step in this investigative process is of course the initial prediction of G4 folding. This is almost exclusively based on the computerized identification of potential G4 (PG4) sequences using a specific search algorithm (or close derivatives thereof) for the G_x_-N_1__–7_-G_x_-N_1__–7_-G_x_-N_1__–7_-G_x_, sequence where *x* is ≥ 3 and *N* corresponds to any of the 4 nt (A, G, C and T or U) (1,2). These algorithm criteria were developed using results from various *in vitro* experiments but primarily from DNA G4 folding studies. Several discrepancies concerning PG4s identified via this algorithm have been reported in recent years. Certain sequences not fulfilling all of the algorithm’s criteria were indeed shown to fold into G4s, i.e. to be false negatives. The DNA G4 reported for the CEB25 minisatellite is a good example of one such false negative (9). Because of its central 9-nt loop, the algorithm did not predict it would form a G4. It has also been shown that DNA PG4s including single-nucleotide loops 1 and 3 support the presence of a large loop 2 of up to 21 nt in length (10). Similarly, RNA G4s including loops up to 15 nt long have also been reported to fold into stable G4s, both *in vitro* and *in cellulo* (11) (Rouleau S., Beaudoin JD. and Perreault JP., unpublished data). Recently, Mukundan *et al.* reported the *in vitro* formation of artificial DNA G4s with multiple bulges involving discontinuous guanine tracks (G-tracks), i.e. differing from the standard algorithm (12). Another guanine-rich RNA sequence ignored by the algorithm was reported to form an atypical G4 bearing discontinuous G-tracks. The high-resolution structure determined for the *sc1* RNA bound to a peptide from the human fragile X mental retardation protein eloquently illustrates both the heterogeneity and complexity of the web of RNA strand interactions involved in G4 folding (13). There are also many reports of false positives, i.e. of PG4s identified via the algorithm that do not to fold into G4s, both *in vitro* and *in cellulo*. One study focusing on human 5′-UTR mRNA G4s reported that several selected PG4s fulfilling all of the algorithm’s requirements were in fact unable to fold into G4s (6) owing to cytosine tracks (C-tracks) located in their flanking sequences, i.e. within 10–15 nt either in 5′ or 3′ of the PG4. It turns out that these C-tracks interacted with the G-tracks of the PG4 sequence, producing alternative secondary structures based on Watson–Crick base pairs. This impaired G4 folding by sequestering key guanines. For each of these non-folding PG4s, substitution mutants bearing adenosines instead of cytosines, to destabilize the inhibitory secondary structure, were shown to successfully fold into G4s (6).

Here, we investigated the folding of a PG4 located in the 3′-UTR of the TTYH1 gene both *in vitro* and *in cellulo*. Data indicate that the presence of multiple cytosines within the PG4’s genomic context inhibits G4 folding. To broaden our investigation of the influence of the PG4’s neighbouring sequences and their impact on G4 folding, we screened multiple biological RNA PG4s. Results permitted the development of a predictive score for G4 folding. This novel scoring system can be used to cure PG4 databases of false-positive candidates. The risks and benefits of our scoring system for the identification of PG4s within genomes and transcriptomes are also discussed.

## MATERIALS AND METHODS

### Bioinformatics

Human 5′- and 3′-UTR databases were derived from sequences obtained from UTRdb (UTRef release 9) (14). PG4 sequences were ascertained using the RNAMotif program (15) and the following algorithm search sequence: G_x_-N_1__–7_-G_x_-N_1__–7_-G_x_-N_1__–7_-G_x_, where *x* is ≥3 and *N* corresponds to any of the 4 nt (A, G, C or U). Only PG4s distanced by a minimum of 10 nt were retained. Data output from the RNAMotif program was exported into Excel file format using various Perl scripts to generate the data sets. Data sets 1 and 2 from 5′- and 3′-UTRs, respectively, are available in the Supplementary Material. Minimum free energy values (Mfe, kcal/mol) were predicted by the RNAfold software from the Vienna RNA Package (16).

The cG score is calculated on a string ‘s’ of length ‘n’ as follows:

‘Gs(i)’ represents the set of all substrings of consecutive ‘Gs’ found in s, and ‘|Gs(i)|’ is the cardinality of this set. Note that all substrings in this set are identical but correspond to different regions of ‘S’ (e.g. the string ‘GGGCGGG’ has 2 ‘GGG’ substrings and thus |Gs (3)| will be 2)
The cG score of string s is then defined as

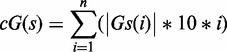

The cC score is calculated in a similar manner:

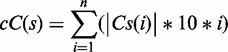

In other words, for a given PG4, a value of 10 is attributed for each G or C, and then, a value of 20 for each doublet (GG or CC), a value of 30 for each triplet (GGG or CCC), and so on. The cG or cC score is the sum of all Gs’ and Cs’ attributed values, respectively. For example, three consecutive Gs will generate a total cG score of 100 because it is counted as three single Gs, two different doublets and one triplet [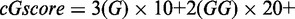 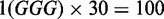], whereas two consecutive Gs have a total cG score of 40 [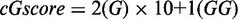 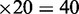].

Finally, the cG/cC score was calculated as the ratio of both scores:

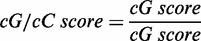

Receiver-operator characteristic (ROC) curves analyses were performed using the GraphPad Prism version 5.02 for Windows, GraphPad Software, San Diego, CA, USA. Briefly, total loop length, Mfe, cG/cC score and QGRS G-score values of each long-context PG4 (12 candidates of the first set) were divided into G4 folding or non-folding categories, based on in-line probing results. Specificity was measured as the fraction of non-folding candidates with prediction parameters inferior to the threshold value. Sensitivity was evaluated as the fraction of G4 folding candidates with a predictive parameter over the threshold value. The threshold was the value midway between a pair of PG4 values. Paired specificities and sensitivities were evaluated for all such threshold values and plotted on a graph where the area under the curve (AUC) is the ability to discriminate between G4 folding and non-folding. An AUC of 0.5 is a random, i.e. non-discriminating value, whereas an AUC of 1 demonstrates perfect discrimination.

### RNA synthesis

The detailed protocol for the analysis of PG4s by in-line probing has been described previously (17). First, double-stranded DNA sequences corresponding to each PG4, and containing the T7 RNA promoter sequence, were prepared. All of the oligodeoxyribonucleotide sequences used are presented under Supplementary Table S1. Two overlapping oligonucleotides (2 µM each, Invitrogen) were annealed and then purified *Pfu* DNA polymerase was used to fill in the gaps in the presence of 5–10% DMSO (Fisher scientific), 2 mM MgSO_4_, 0.2 mM of each dNTP, 20 mM Tris–HCl, pH 8.8, 10 mM KCl, 10 mM (NH_4_)SO_4_ and 0.1% Triton X-100. Full-length double-stranded DNAs were then ethanol-precipitated and the resulting pellet was dissolved in ultrapure water. RNA transcripts were prepared by *in vitro* run-off transcription using purified T7 RNA polymerase (10 µg) in the presence of 20 U of RNase OUT (Invitrogen), 0.01 U of pyrophosphatase (Roche Diagnostics) and 5 mM NTPs in a buffer containing 80 mM HEPES-KOH, pH 7.5, 24 mM MgCl_2_, 2 mM spermidine and 40 mM DTT, all in a final volume of 100 µl. The reactions were incubated at 37°C for 2 h. Fifteen minutes before the end of the incubation, 3 U of DNase RQ1 (Promega) were added. RNAs were then purified by phenol-chloroform extraction and ethanol precipitated before being dissolved in 30 µl of water. RNA products were then fractionated by denaturing (8 M urea) 7–10% (depending of the length of the candidates) polyacrylamide gel electrophoresis (polyacrylamide gel electrophoresis; 19:1 acrylamide:bisacrylamide) using a 45 mM Tris–borate, pH 7.5, 1 mM EDTA running buffer. RNA product bands were visualized by ultraviolet shadowing, and those of the correct sizes were excised from the gel and the transcripts eluted overnight at 4°C in elution buffer (1 mM EDTA, 0.1% sodium dodecyl sulphate and 0.5 M ammonium acetate). RNA PG4s were then ethanol-precipitated, dried and dissolved in water. Concentrations were determined by spectrophotometry at 260 nm using a GE Nanovue spectrometer.

### Circular dichroism spectroscopy and thermal denaturation analysis

Circular dichroism (CD) experiments were performed using 4 µM of the relevant RNA sample in 50 mM Tris–HCl (pH 7.5) buffer either in the absence of salt, or in the presence of 100 mM of either LiCl, NaCl or KCl. Before taking the CD measurement, each sample was heated at 70°C for 5 min and then slow-cooled to room temperature over a 1-h period. Experiments were performed using a Jasco J-810 spectropolarimeter equipped with a Jasco Peltier temperature controller in a 1-ml quartz cell cuvette with a path length of 1 mm. CD scans, ranging from 220 to 320 nm, were recorded at 25°C at a scanning speed of 50 nm min^−^^1^ with a 2-s response time, 0.1 nm pitch and 1 nm bandwidth. All CD data represent the average of three wavelength scans. Subtraction of the buffer was not required, as control experiments performed in the absence of RNA showed negligible curves. For thermal denaturation analysis, samples were heated from 25 to 90°C at a controlled rate of 1 min^−^^1^ and a 264 nm CD peak was monitored every 0.2 min to obtain the CD melting curves. Melting temperature values (T_m_) were calculated using ‘fraction folded’ (θ) versus temperature plots (18).

### RNA labelling

Before radioactive 5′-end-labelling, 50 pmol of purified RNA transcripts were dephosphorylated using 1 U of antarctic phosphatase (New England BioLabs) in a 10-µl final volume reaction containing 50 mM Bis-Tris-propane, pH 6.0, 1 mM MgCl_2_, 0.1 mM ZnCl_2_ and 20 U RNase OUT (Invitrogen). Reactions were incubated at 37°C for 30 min and the enzyme was then inactivated by incubating at 65°C for 7 min. For the 5′-end-labelling reaction itself, dephosphorylated transcripts (10 pmol) were incubated at 37°C for 1 h in the presence of 3 U of T4 polynucleotide kinase (USB), 3.2 pmol of [γ-^32^P] ATP (6000 Ci/mmol; New England Nuclear), 20 U of RNase OUT (Invitrogen) and in a buffer with final concentrations of 50 mM Tris–HCl, pH 7.6, 10 mM MgCl_2,_ 10 mM β-mercaptoethanol, all in a final volume of 10 µl. Labelling reactions were stopped by the addition of 10 µl of formamide dye buffer [95% formamide, 10 mM EDTA, 0.025% bromophenol blue and 0.025% xylene cyanol (XC)]. Radiolabelled transcripts were fractionated by 7–10% denaturing polyacrylamide gel electrophoresis, and the bands were visualized by autoradiography. Those bands corresponding to transcripts of the correct sizes were excised from the gel and RNAs recovered and purified as described above. Purified 5′-end-labelled transcripts were dissolved in 30 µl of nanopure water, and radioactivity (total cpm) was measured using the Cerenkov method and Bioscan QC-2000 radioactivity counter.

### In-line probing

Trace amounts (50 000 cpm, <1 nM) of each 5′-end-labelled RNA were heated to 70°C for 5 min and then slow-cooled to 25°C (≈1 h) in a buffer containing 20 mM lithium cacodylate (pH 7.5) and either in the absence of salt, or in the presence of 100 mM of LiCl, NaCl or KCl (depending on the condition tested), all in a final volume of 10 µl. After slow-cooling, the volume of each reaction was adjusted to 100 µl to obtain final concentrations of 20 mM lithium cacodylate (pH 8.5), 20 mM MgCl_2_ and 100 mM of LiCl, NaCl or KCl. Reactions were incubated for 40 h at 25°C to allow for self-cleavage of the RNA to occur. After incubation, samples were ethanol-precipitated in presence of glycogen, ethanol-washed and dissolved in 10 µl of formamide dye buffer (that contained only XC as marker dye). An alkaline hydrolysis ladder was prepared with 50 000 cpm of the 5′-end-labelled transcript that was dissolved in 5 µl of water. One microliter of 1 N NaOH was added, and reactions were then incubated for 1 min at 25°C. Reactions were stopped by addition of 3 µl of 1 M Tris–HCl, pH 7.5, and then ethanol precipitated and dissolved in 10 µl of formamide loading dye (that contained only XC as marker dye). RNase T1 ladder was prepared by the addition of 0.6 U of RNase T1 (Roche Diagnostic) to 50 000 cpm of 5′-end-labelled transcript that was dissolved in 9 µl of buffer containing 20 mM Tris–HCl, pH 7.5, 10 mM MgCl_2_ and 100 mM LiCl. Reactions were incubated for 2 min at 37°C, and then stopped by the addition of 20 µl of formamide dye buffer (that contained only XC as marker dye). Both samples and ladders were transferred to new eppendorf tubes and radioactivity (total cpm) measured using a Bioscan QC-2000 radioactivity counter. Both samples and alkaline hydrolysis ladder were then diluted to obtain equal amounts of radioactivity (cpm) for each loading sample, and ∼two-third of these amounts of radioactivity for RNase T1 ladders. Samples and ladders were then fractionated by denaturing (8 M urea) 7–10% (depending on candidate size) polyacrylamide gel electrophoresis. Gels were dried and exposed overnight to a phosphoscreen. Bands were visualized by phosphorimaging using a Typhoon Trio instrument (GE Healthcare). Quantitative analyses of the bands were performed using the SAFA software (19). Two independent in-line probing experiments were performed and quantified for each candidate. Results are presented as one representative gel and a bar graph of the means and standard deviations of K^+^/Li^+^ intensities’ ratios obtained from both experiments. A candidate is considered positive for G4 folding if the K^+^/Li^+^ ratio is ≥2 (or significantly different from 1) for at least 2 nt predicted to be located in the loops and/or immediately next to the first or last G-track. Banding pattern must differ from that of the mutated version abolishing G4 folding. Characteristics needed to be reproducible in at least two independent experiments.

### Plasmid constructions

The sequences of the full-length 3′-UTRs of LRP5 and TTYH1 were obtained from the NCBI database and correspond to the following GenBank Accession numbers: LRP5, NM_002335; TTYH1, NM_020659. The 3′-UTRs were reconstituted *in vitro* by the filling in of multiple overlapping oligonucleotides and various polymerase chain reaction steps. The other plasmid constructs (TTYH1 + pAS, TTYH1 LRP5-pAS, LRP5 Ty PG4) were obtained by oligonucleotide-directed mutagenesis (see Supplementary Tables S3 and S4 for both the detailed sequences and the list of the oligonucleotides used). Both the wild-type (WT) and a G/A-mutant were synthesized for all 3′-UTR sequences. C/A- and GC/AA-mutant versions of the TTYH1 3′-UTR were also synthesized. The positions of the mutations were identical to those used for the *in vitro* in-line probing experiments. The different 3′-UTR constructs were inserted into the *Xba*I and *BamH*I restriction sites in the pGL3 plasmid vector (Promega). The correct insertion of each construct was confirmed by DNA sequencing.

### Cell culture

HEK293T cells were cultured in Dulbecco’s Modified Eagle Medium supplemented with 10% fetal bovine serum, 1 mM sodium pyruvate and an antibiotic-antimycotic drug mixture (all from Wisent) at 37°C in a 5% CO_2_ humidified incubator.

### Dual luciferase assays

HEK293T cells were seeded in either 24- (1.7 × 10^5^) or 48-well plates (8.5 × 10^4^) 24 h before transfection. Cells were co-transfected with 400 ng of the specific pGL3 plasmid construct (Firefly luciferase, Fluc) and 100 ng of the pRL-TK control vector (Renilla luciferase, Rluc) (Promega) using Lipofectamine 2000 (Invitrogen) in Opti-MEM (Gibco) lacking the antibiotic-antimycotic mixture. Twenty-four hours after transfection, cells were lysed using passive lysis buffer (Promega). Fluc and Rluc activities were measured using the Dual-luciferase Reporter Assay kit (Promega) according to the manufacturer’s protocol on a GloMax 20/20 Luminometer (Promega). For each condition, the Fluc value was normalized by dividing it by the Rluc value. Ratios of normalized WT version to normalized G/A-mutant version were calculated. Results are presented as means and standard deviations of a minimum of three independent experiments for each candidate.

## RESULTS AND DISCUSSION

### The PG4 sequence in the 3′-UTR of TTYH1 folds *in vitro*

**Figure 1.**
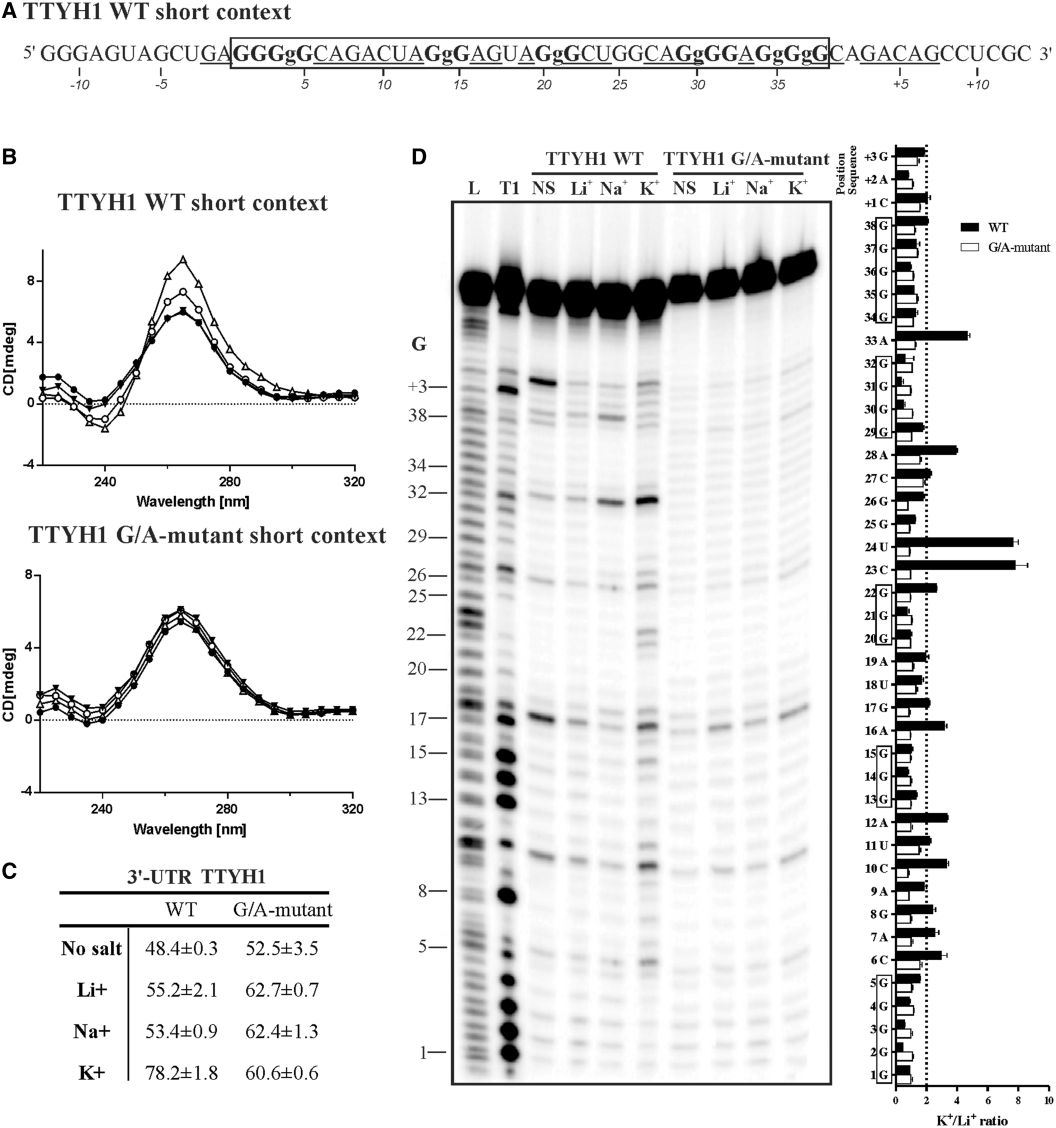
*In vitro* analysis of the TTYH1 WT PG4. (**A**) Sequence of the TTYH1 WT PG4 surrounded by a short genomic context of 12–13 nt on both sides. The predicted PG4 sequence is composed of five G tracks and is boxed. The guanines involved in the G tracks are in bold. Lower case guanines (g) stand for those that were mutated to adenines in the G/A-mutant version. Nucleotides displaying greater cleavage accessibility in the in-line probing experiments are underlined. (**B**) CD spectroscopy analysis performed in the absence of salt (filled circles), or in presence of 100 mM of either lithium (Li^+^, filled triangles), sodium (Na^+^, circles) or potassium (K^+^, triangles). *Top panel:* The WT version displays a negative peak at 240 nm and a positive one at 264 nm, which is characteristic of folding for a G4 with parallel topology. *Lower panel:* The equivalent spectrum for the G/A-mutant version in which certain guanines of the G tracks were mutated to adenines to abolish any possible G4 folding. (**C**) Thermal denaturation analysis of both the WT and the G/A-mutant versions either in the absence of salt, or in the presence of 100 mM of Li^+^, Na^+^ or K^+^. Melting temperature values (T_m_) were calculated using ‘fraction folded’ (θ) versus temperature plots (18). (**D**) In-line probing analysis of both the TTYH1 WT and the G/A-mutant versions in either the absence of salt (No salt, NS), or in the presence of 100 mM of Li^+^, Na^+^ or K^+^. Bar graph represents the K^+^/Li^+^ intensity ratios of two independent experiments. Error bars represent standard deviation.

It has been previously demonstrated that G4s present in 3′-UTRs of mRNAs can stimulate polyadenylation when they are located downstream of a polyadenylation site (7,20). However, this has only been demonstrated in three distinct cases, i.e. LRP5, FXR1 and P53. To provide additional physical support for this phenomenon, the folding of the G4 motif within the TTYH1 gene (GenBank Accession number: GI 319803129, NM_020659) was investigated. The product of this gene is a calcium-independent volume-sensitive chloride channel (21). This candidate was retrieved from a database that included all PG4s found in human 3′-UTR sequences using the classic algorithm search sequence G_x_-N_1__–7_-G_x_-N_1__–7_-G_x_-N_1__–7_-G_x_, where x ≥ 3 and N is any nucleotide (A,C,G or U) (1,2,7). When considering the PG4 sequence alone, folding appeared highly probable (Figure 1A). The sequence bears 5 G-tracks and a few (1–7) intercalated nucleotides providing multiple possibilities of G4 conformations by various G-track combinations. This was not possible in the three cases (LRP5, FXR1 and P53) reported previously. The TTYH1 PG4 candidate was also chosen because its 3′-UTR was relatively short (i.e. 348 nt), thereby circumventing a number of potential difficulties in the cloning step required for subsequent *in cellulo* investigations.

Initially, both G4 folding and topology were assessed by CD spectroscopy. A positive peak at 264 nm and a negative peak at 240 nm are characteristic of a parallel G4 topology (22). A WT sequence exceeding the TTYH1 PG4 in length by 12 nt in 5′ and 13 nt in 3′ was studied to assess G4 folding *in vitro* (see Figure 1A). Sequences at both extremities preserved some of the natural 3′-UTR genomic context (7,23). A G/A-mutant version bearing six substitution adenines, i.e. at least one in each G-track, which prevented G4 folding, was also *in vitro* transcribed for use as a negative control (see Figure 1A). Initially, CD spectrums were recorded either in the absence of salt or in the presence of 100 mM of LiCl. No significant difference was observed between WT and G/A-mutant versions, confirming that the G4 folding did not occur under these conditions (see Figure 1B). The spectrum was then recorded in the presence of 100 mM of either NaCl or KCl, i.e. conditions that support G4 folding. Significant changes in both the 240 and 264 nm peaks were detected only for the WT sequence, confirming that G-quartets were stabilized by the monovalent cations, especially K^+^ (Figure 1B). The conclusions drawn from the CD experiments received additional physical support from thermal denaturation analysis, where the melting temperature (T_m_) for the WT version was found to be higher under the K^+^ condition. In contrast, results for the G/A-mutant showed no difference between the salt conditions (Figure 1C).

Further *in vitro* support was obtained from in-line probing of both WT and G/A-mutant PG4s. This simple technique, which uses only trace amounts of radiolabelled RNA molecules (<1 nM), and thus favours intramolecular G4 folding, relies on the spontaneous cleavage of RNA under physiological conditions (17). Flexible regions, such as single-stranded nucleotides, are relatively more prone to cleavage. In G4s, the connecting loops between G-tracks are typically flexible. TTYH1 transcripts were ^32^P-5′-labelled before being incubated for 40 h at 25^°^C in the presence of MgCl_2_ and either in the absence of salt, or in the presence of 100 mM of LiCl, NaCl or KCl. Resulting samples were fractionated by denaturing (8 M urea) gel electrophoresis. Several WT transcript bands from the KCl sample (a condition that is a favourable for G4 formation) were relatively more intense than corresponding bands from the other samples (Figure 1D). Illustrating band intensity data by means of a bar graph displaying variations as K^+^/Li^+^ ratios showed that nucleotides exhibiting the highest susceptibility to hydrolysis were located between G-tracks and immediately 5′ and 3′ of the first and last G-tracks, respectively, a situation that is typical of G4 folding (see also the underlined residues in the TTYH1 PG4 sequence, Figure 1A) (17). Thus, three distinct and complementary methods show that the TTYH1 PG4 folds *in vitro* in the presence of K^+^.

### Neighbouring C-rich sequences affect G4 folding

**Figure 2.**
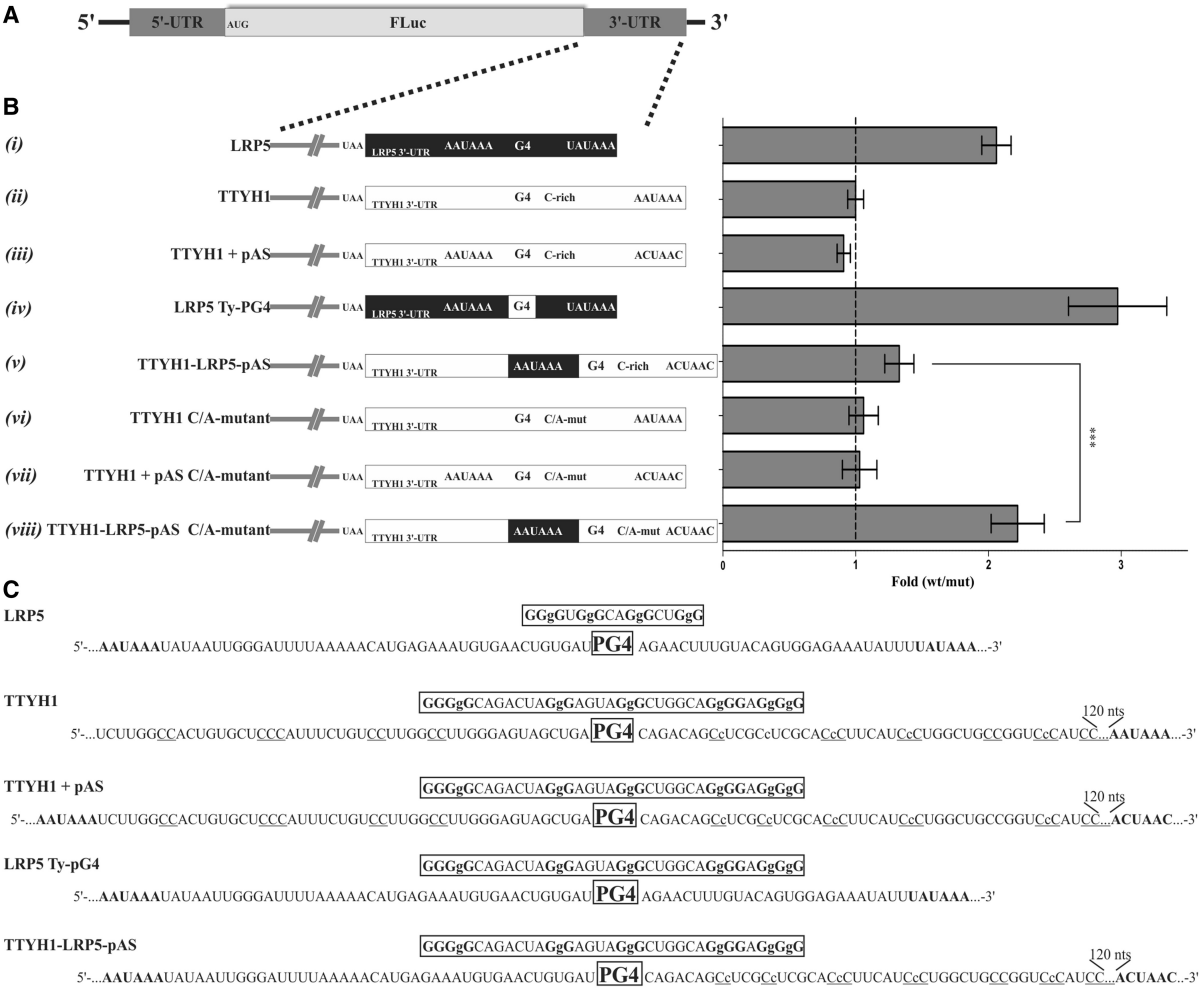
Luciferase assays measuring the effects of the 3′-UTR G4s on the stimulation of gene expression. (**A**) Schematic representation of the firefly luciferase reporter gene construct transfected into HEK293T cells. Full sequences of all of the constructs used are listed in Supplementary Table S3 (**B**) Left panel: Schematic representation of all of the full-length 3′-UTRs used. The black and white rectangles represent the sequences of LRP5 and TTYH1, respectively. Constructs with ‘pAS’ had their canonical polyadenylation site (AAUAAA) abolished through mutation to ACUAAC. Those identified as C/A-mutant had several of cytosines located downstream of the G4 mutated to adenines to abolish the C tracks. Right panel: Gene expression levels of the different constructs, as measured at the protein level with the firefly luciferase assay, are represented as a fold difference of the value obtained for the G4 WT version divided by that obtained for the G4 mutant version (in which key guanines of the G4 were mutated to adenines to abolish G4 folding). Error bars (standard deviations) were calculated with the results of at least three independent experiments. *P*-values were evaluated with a two-tailed unpaired Student *t*-test. ***P < 0.0001. (**C**) Partial sequences of the different 3′-UTR constructs. The PG4 region is boxed, guanines of the G tracks and the polyadenylation signal (pAS) are shown in bold and C-rich regions are underlined. Lowercase cytosines (c) are those mutated to adenines in the C/A-mutant versions.

The TTYH1 PG4 was further analysed to assess its ability to fold *in cellulo*. The full-length 3′-UTR sequence was cloned downstream of a firefly luciferase reporter gene and analysed using a dual luciferase system (see Figure 2A and the ‘Materials and Methods’ section). HEK293T cells were co-transfected with plasmids expressing both the firefly and renilla luciferases (for normalization), grown for 24 h an then harvested for the performance of the luciferase assays. As 3′-UTR G4s are known to stimulate gene expression, a 2-fold difference in protein synthesis of the WT over G/A-mutant would suggest G4 folding (see Figure 2B). The 3′-UTR of LRP5 was used as a positive control and showed an ∼2-fold difference, reflecting a higher level of protein synthesis during G4 folding [Figure 2B(i)]. A previous report suggested that the 3′-UTR LRP5 PG4 folded into a G4 *in cellulo*, stimulating polyadenylation at a non-canonical upstream site (7). For the TTYH1 WT PG4, protein synthesis remained unchanged, suggesting an absence of G4 folding in the context of the native full-length sequence [Figure 2B(ii)]. The TTYH1 PG4 sequence commences at the 120th nt position of the 3′-UTR and bears a canonical polyadenylation signal (pAS) commencing at the 168th nt position downstream of the g-quadruplex. The absence of any significative difference in luciferase expression for the TTYH1 WT construct may result from the fact that TTYH1 lacks an upstream pAS as opposed to LRP5 (see the comparative schematics of the 3′-UTR architectures under Figures 2B(i) and (ii)). To verify this hypothesis, a pAS was inserted 49 nt upstream of the TTYH1 PG4, i.e. in a position analogous to its location in the LRP5 sequence [TTYH1 + pAS, Figure 2(iii)]. In addition, the canonical signal was mutated to force polyadenylation to occur at a position potentially stimulated by the PG4, once again to mimic the effect of the LRP5 G4. No significant difference was observed with this construct [Figure 2B(iii)], suggesting that either the TTYH1 PG4 remained unfolded *in cellulo*, or that a required cofactor was lacking. The LRP5 PG4 sequence was then substituted for the TTYH1 PG4 sequence while conserving the remaining LRP5 3′-UTR intact [i.e. LRP5 Ty-PG4; Figure 2B(iv)]. This construct displayed a significant 3-fold increase in protein synthesis compared with the LRP5 WT, indicating that the TTYH1 PG4 folded *in cellulo* and stimulated polyadenylation more efficiently than the LRP5 G4.

Results presented above suggested that unaltered protein synthesis for TTYH1 PG4 within its natural 3′-UTR context may be attributable to the composition of the neighbouring sequences. To further investigate this possibility, several constructs were engineered. A first construct was synthesized by inserting not only the 6 nt of the LRP5 pAS of the TTYH1 + pAS version, but also additional nucleotides located between this pAS and the first G-track of the LRP5 PG4, creating the TTYH1-LRP5-pAS construct [Figure 2B(v)]. Thus this construct would contain potential *cis*-regulating elements important for polyadenylation, such as the U-rich region located downstream of the cleavage site, as well as both the primary and secondary structures surrounding it. However, still no significant difference in protein synthesis was observed [Figure 2B(v)]. Alternatively, we hypothesized that the absence of effect might be due to folding hindrance attributable to C-tracks located (13–50 nt) mainly downstream but also upstream of the PG4. An earlier study revealing a large number of neighbouring cytosine residues in a PG4 retrieved in the 5′-UTR region of three mRNAs impaired for G4 folding (6), suggested that C-rich regions could compete with G-tracks to form Watson–Crick base pairs, thereby hindering G4 folding. Neighbouring cytosine residues were, however, not part of the short transcript studied previously *in vitro*, or the LRP5 Ty-PG4 construct. We then replaced certain cytosine residues by adenines in the TTYH1 and TTYH1 + pAS C/A-mutants [Figure 2B(vi) and (vii), respectively]. Still no effect was observed with either of these C/A-mutants. Importantly, both constructs were deprived of essential *cis*-regulatory elements. A pAS was also absent from the TTYH1 C/A-mutant. Next, we engineered a TTYH1-LRP5-pAS C/A-mutant version including both *cis*-regulatory elements and a pAS [Figure 2B(viii)]. This construct exhibited a significant 2-fold increase in luciferase expression. Taken together, these data suggest that a series of cytosines located as far as 20–50 nt from the PG4 can significantly influence G4 folding *in cellulo*, demonstrating that regulation of G4 folding is far more complex than what has been previously reported (6,7).

### In-line probing of TTYH1-derived transcripts

**Figure 3.**
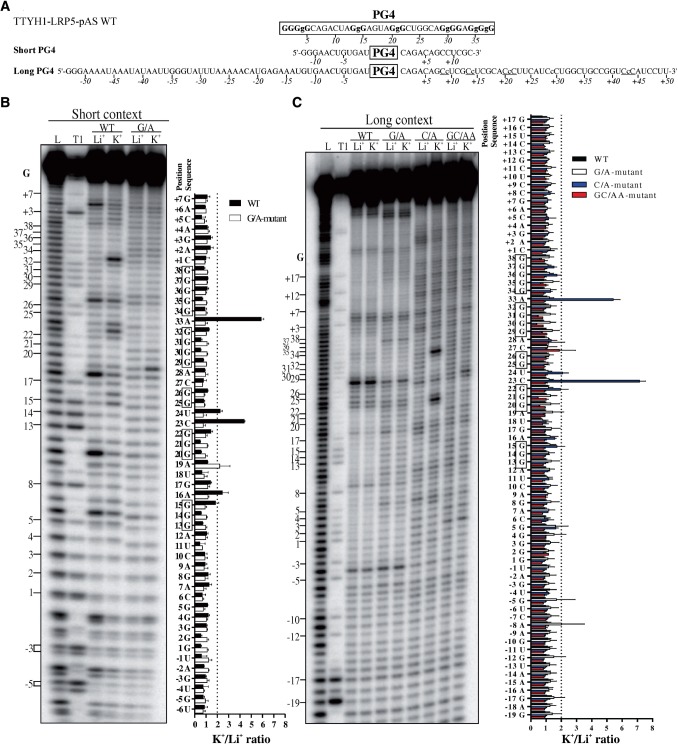
In-line probing and quantitative analysis of structures adopted by the TTYH1-LRP5-pAS PG4 candidate in both short and long genomic contexts. (**A**) Sequences of the TTYH1-LRP5-pAS PG4 candidate with its short (∼13 nt each side) and long (∼50 nt each side) genomic contexts. The predicted PG4 sequence is boxed. Guanines of the G tracks are shown in bold. Nucleotide positions are indicated underneath the sequence and ‘+’ and ‘−’ indicate whether the genomic nucleotide is located downstream or upstream of the PG4, respectively. Lowercase guanines (g) and cytosines (c) represent those mutated to adenines in the G/A- and C/A-mutant versions, respectively. C tracks are underlined. (**B**) Autoradiogram of a 10% denaturing (8 M urea) polyacrylamide gel of the in-line probing of both the WT and G/A-mutant versions of the TTYH1-LRP5-pAS in their short genomic contexts. (**C**) Autoradiogram of a 7% denaturing (8 M urea) polyacrylamide gel of the in-line probing of the WT, G/A-, C/A- and GC/AA-mutant versions of the TTYH1-LRP5-pAS in the long genomic context. For both genomic context lengths (B and C), in-line probings were performed in presence of 100 mM of either LiCl (Li^+^) or KCl (K^+^). Positions of the guanines are indicated on the left of each gel. L and T1 are the alkaline hydrolysis and ribonuclease T1 mapping lanes, respectively. Bar graphs (right portions of panels B and C) represent the quantitative analysis of in-line probing and show, for each band, the intensity values in the K^+^ condition divided by that in the Li^+^ condition. The dotted line corresponds to the threshold of two that denotes the nucleotides with higher accessibilities to in-line cleavage in the presence of K^+^ (i.e. the nucleotides located in the G4 loop). G tracks predicted to be implicated in G4 folding are boxed. Black, white, blue and red stand for the WT, G/A-mutant, C/A-mutant and GC/AA-mutant versions, respectively, coloured figure online. Error bars are standard deviations as calculated from two independent experiments. The G4 is formed only in the cases of the short WT context and the long C/A-mutant version contexts.

To further investigate the influence of C-rich regions on TTYH1 G4 folding, *in vitro* in-line probing experiments were performed. A short and a long transcript corresponding to the TTYH1-LRP5-pAS WT were synthesized. A TTYH1-LRP5-pAS C/A-mutant was synthesized in the long version only. In the short version, the PG4 was flanked in 5′ and 3′ by 9 and 13 nt, respectively. In the long version, the PG4 was flanked on each side by a 50-nt sequence found in the mRNA (Figure 3A). For all transcripts, a G-track G/A-mutant version was also engineered for purposes of comparison between the presence and absence of PG4. An autoradiogram of a representative in-line probing for the TTYH1-LRP5-pAS transcript is illustrated on the left panel of Figure 3B. The right panel of Figure 3B shows the quantitative analysis of two independent experiments in the form of a bar graph representing K^+^/Li^+^ intensity ratios. Nucleotides with a ratio value superior to the arbitrary threshold of 2 were considered as being accessible under conditions favouring G4 folding (17). Band intensities increased for WT version residues located in single-stranded regions adjacent to G-tracks on G4 folding and solely in the presence of K^+^. These residues correspond to nucleotides A_16_, C_23,_ U_24_ and A_33_. While the TTYH1-LRP5-pAS short transcript folded into a G4, the same PG4 located within the long transcripts displayed no significant difference in banding patterns compared with WT and G/A-mutant versions, regardless of whether incubation was performed in the presence of LiCl or KCl (Figure 3C). These results indicated that the PG4 did not fold differently in the presence of K^+^. Conversely, the C/A-mutant version bearing five substitute adenosines between positions 9–43 displayed two additional bands of greater intensity in the presence of KCl, compared with both WT and G/A-mutant versions (Figure 3C). Both of these corresponded to nucleotides C_23_ and A_33_, as was the case for the short version. These data show that mutating the C-track to hinder potential GC Watson–Crick base pairs was sufficient to favour G4 folding. Taken together, these results confirm that C-tracks located up to 20–50 nt from the PG4 can prevent G4 folding, and that folding can be rescued where C-tracks are either completely absent as in the short context version, or replaced by adenines as in the C/A-mutant version.

### Assessing the PG4 genomic context

**Figure 4.**
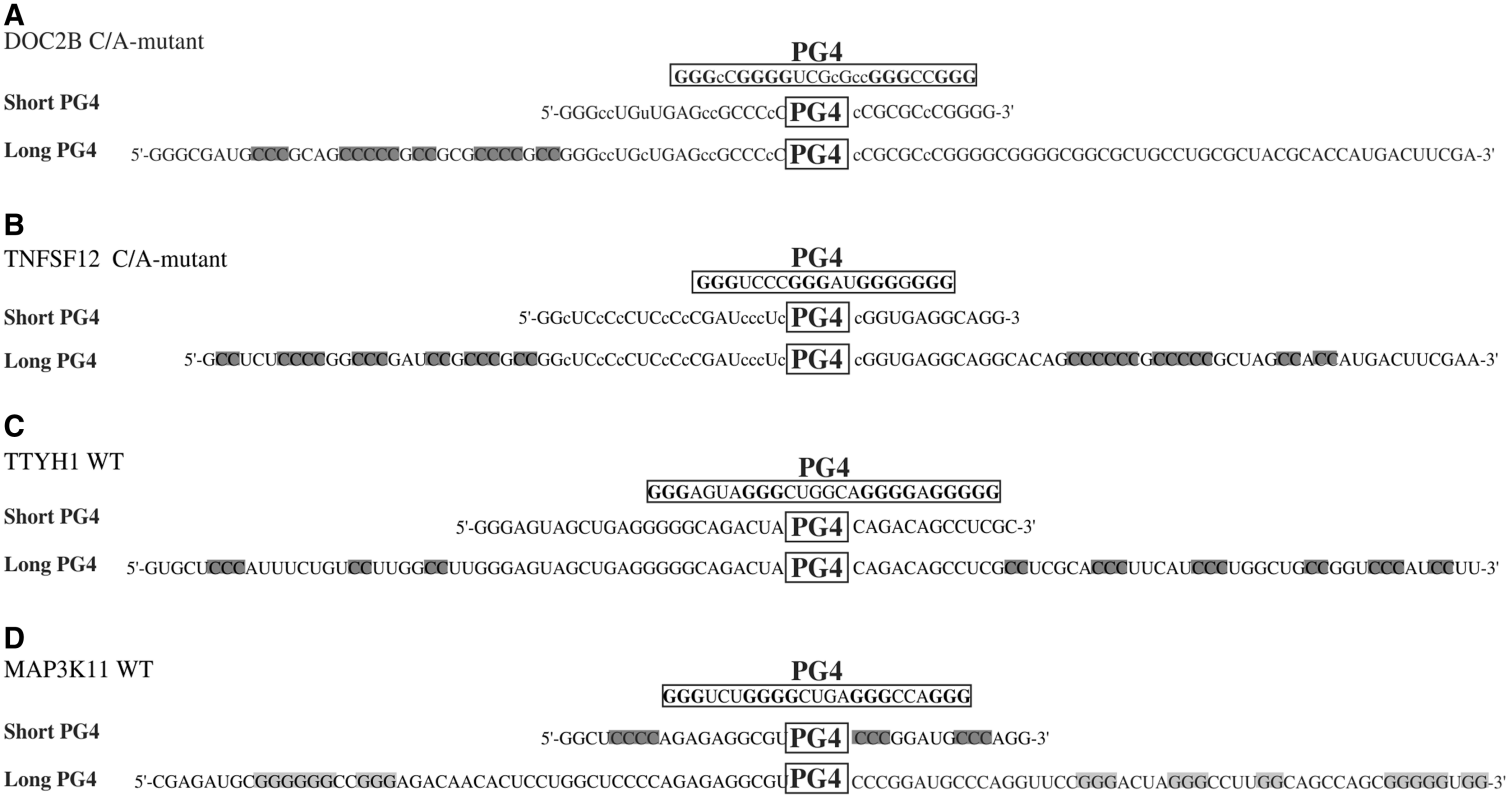
Sequence analysis of the genomic context of non-folding PG4s. (**A**) DOC2B C/A-mutant; (**B**) TNFSF12 C/A-mutant; (**C**) TTYH1 WT; and (**D**) MAP3K11 WT. The PG4 sequences predicted by the algorithm are boxed. Guanines involved in the G tracks are shown in bold. Sequences of both the short and long genomic context versions are presented. Lower case cytosines (c) represent those mutated to adenines in the C/A-mutants. Inhibitory tracks of cytosines in the genomic context version are highlighted in dark gray. Enhancing tracks of guanines are highlighted in pale gray.

Results with the TTYH1-LRP5-pAS transcript unambiguously showed that PG4 neighbouring sequences have a significant impact on G4 folding. Next, to broaden the scope of this study, we investigated 11 other PG4s from human 5′- and 3′-UTRs, as well as three C/A-mutant versions of 5′-UTR PG4s for which mutations were shown to be required for G4 folding (6). All of these candidates were previously characterized by in-line probing as part of short transcripts bearing ∼15 nt in 5′ and 3′ of the PG4 (4,6). Five variants of the TTYH1-derived constructs were also considered, for 19 candidates. In-line probing was repeated using larger transcripts in which both WT and G/A-mutant versions of PG4s were flanked by ∼50 nt on both sides. The resulting bar graphs for each candidate are presented in Supplementary Figures S1–S12, and a data compilation is given in Table 1. Shaded candidates are those that do not fold into G4s. Thirteen out of the nineteen candidates supported G4 folding of the longer transcripts in the presence of KCl. It is noteworthy that results obtained with both short and long transcripts are in agreement for most of the candidates. In other words, PG4s folded regardless of the size of neighbouring sequences. However, this was not so for four of the candidates, i.e. the DOC2B and TNFSF12 C/A-mutants, and the TTYH1 and MAPK3 WTs (Table 1). Further analysis of the primary sequence of each candidate provided an interesting explanation (Figure 4A–D). The short transcripts of the DOC2B and TNFSF12 C/A-mutants and the TTYH1 WT all support G4 folding in the presence of KCl, but their longer counterparts do not. The extended sequences of these candidates included several C-tracks that most likely form GC Watson-Crick base pairs with residues of the G-tracks, thereby preventing G4 folding (see Figure 4A–C). Some of the possible inhibitory cytosines located in close proximity to the PG4 were replaced by adenines in both the DOC2B and TNFSF12 C/A-mutants. However, cytosines located farther than 15 nt away from the PG4 can still base pair with the guanines from the G-tracks. The MAP3K11 PG4 WT candidate is slightly different, but respects the previous logical assumption (Figure 4D). For this candidate, the short transcript was unable to fold into a G4, most likely due to C-tracks adjacent to the PG4 competing to form inhibitory Watson–Crick base pairs. The longer transcript, however, bore several additional G-tracks, which probably interacted with C-track residues, thereby releasing PG4 and allowing it to fold into a G4 in the presence of KCl. This hypothesis is supported by RNAfold (16) secondary structure predictions for both short and long transcripts (Supplementary Figure S27). Although for most PG4s studied, the length per se of neighbouring sequences did not impact G4 folding, results for four of the candidates point to the inherent complexity of neighbouring sequences as a key issue that must be considered in the accurate prediction of biologically relevant G4 motifs.

**Table 1.** Characteristics of selected PG4 candidates

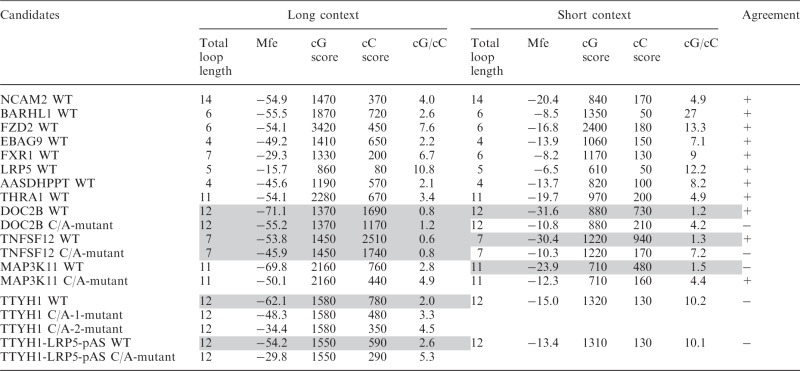

Shaded entries indicate those candidates that cannot fold into G4.

### Determining a predictive parameter of G4 folding

**Figure 5.**
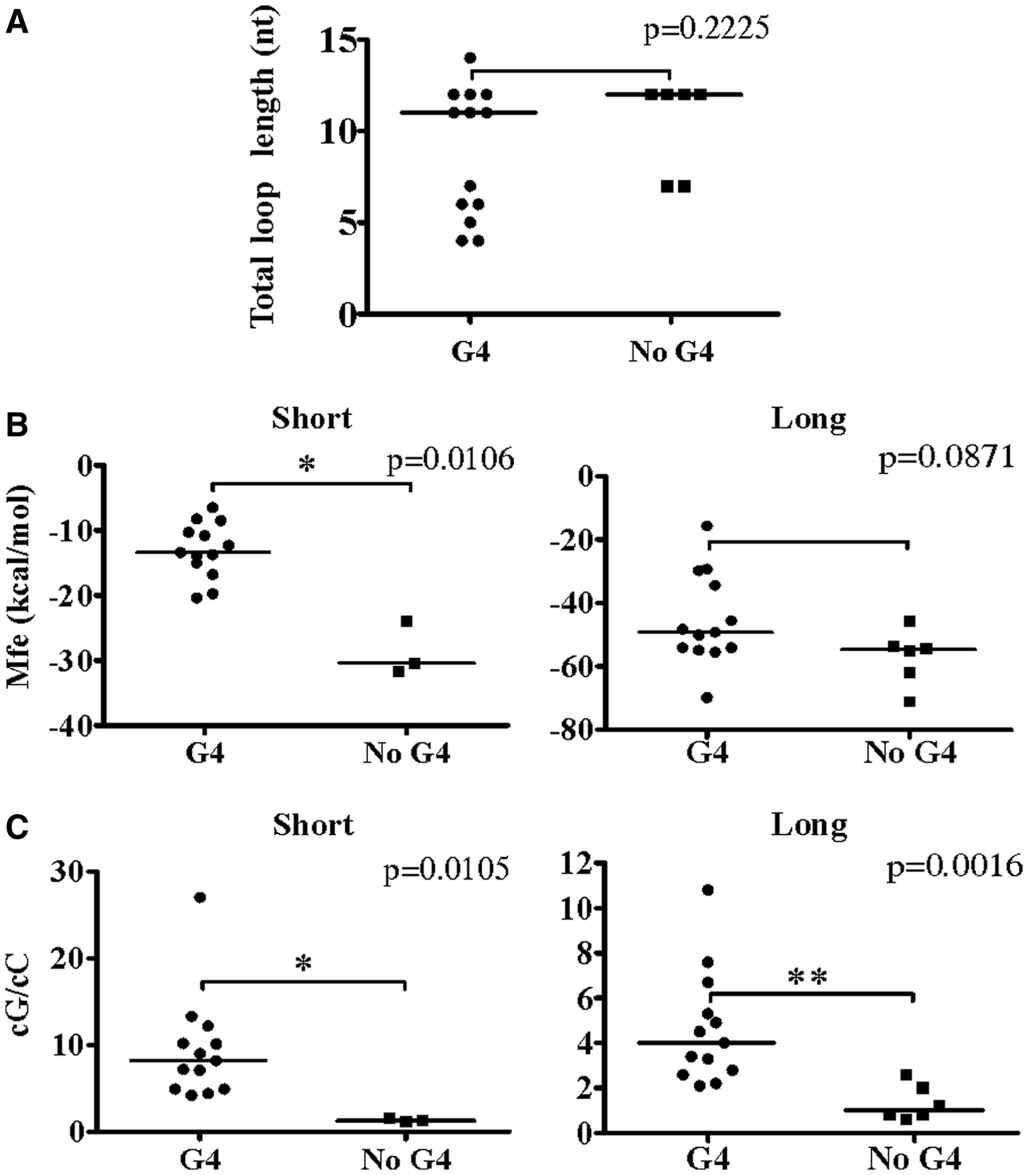
Comparison of the different predictive values of G4 folding for both the short and long genomic context PG4 candidates. (**A**) Predicted total loop length; (**B**) Mfe, kcal/mol, predicted by the RNAfold software from the Vienna RNA Package (16); and (**C**) calculated cG/cC score. Horizontal lines represent the median for each group. *P*-values were evaluated with a Mann–Whitney test. *P < 0.05 **P < 0.01.

Next, we sought to identify a reliable G4 folding predictive value tested against the data obtained for our set of characterized PG4s. Such a value should be able to indicate whether the genomic environment of a given PG4 is favourable to G4 folding. One of the most frequently used criteria for evaluating PG4 stability, and thus G4 folding probability, is the total loop length. For both DNA and RNA G4s, longer loops are associated with relatively less stable free energies (ΔG°) and melting temperatures (Tm) (10,24,25). Accordingly, the lower the total loop length, the more likely the G4 folding. The total loop length for a given PG4 was simply calculated as the sum of the nucleotides present in each of its three loops (Table 1). Surprisingly, for the set of PG4s characterized in this study, total loop length was not a relevant indicator of G4 folding (Figure 5A). No significant difference in total loop length was observed between the folding and non-folding sequences. That said, the majority of PG4s with lower predicted total loop length folded into G4s. However, so did many of the PG4s with higher total loop length, e.g. NCAM2 WT, TTYH1 C/A-1 and -2 mutants. Furthermore, TNFSF12 WT and C/A-mutant sequences, which had relatively lower total loop length, did not fold into G4s. These results are in agreement with those of a previous study performed with human DNA promoter G4s showing that G4 stability did not correlate with loop length (26). For all of these reasons, total loop length did not appear to be a suitable predictive parameter of G4 folding.

As pointed out both here, and in previous reports (6,23,27), the genomic context of a PG4 may influence its folding. It is reasonable to hypothesize that if the genomic context of a given PG4 makes it prone to multiple and strong Watson–Crick base pair–based secondary structures, that this could hinder G4 folding. The thermodynamic stability of an RNA secondary structure can be conveniently estimated using prediction software such as RNAfold from the Vienna RNA Package (16). The software version used, however, considers only Watson–Crick and wobble base pair formation, and therefore could not predict G4 folding. This software provides a Mfe for each predicted structure. According to the formulated hypothesis, a structure with a lower predicted Mfe would be less favourable to G4 folding because the stable Watson-Crick–based secondary structure should form faster than the G4 motif. The secondary structures of both short and long transcripts were predicted using RNAfold, and the Mfe values for the most stable structures were compiled and analysed together (see Table 1 and Figure 5B). For short transcripts, this value was an excellent indicator of G4 folding and non-folding (*P* = 0.0106). However, no significant differences were observed between the Mfe values for both folding and non-folding longer transcripts (*P* = 0.0871). It seems that when considering the longer transcripts, the possibilities of multiple secondary structures increases substantially. Consequently, the use of the Mfe as a predictor of G4 folding within any given transcript is not always valid.

In the absence of a suitable predictive parameter of G4 folding for long RNA transcripts, we attempted to identify one. By definition, G4 folding requires multiple G-tracks in a given sequence. However, there are recent examples of G4 with discontinuous G-tracks or with G-tracks bearing only two consecutive Gs (12,28). Nonetheless, these guanines must be primarily single-stranded, or otherwise sufficiently available, to interact with each other to fold into a G4. Conversely, consecutive cytosines (Cs) have been shown to potentially impair G4 folding, most likely due to pairing with consecutive Gs within a stable Watson–Crick base-paired structure. Following this rationale, separate consecutive G (cG) and consecutive C (cC) scores were considered (see ‘Materials and Methods’ section). Briefly, a score of 10 was attributed for each single G or C, a score of 20 for each doublet GG or CC, 30 for each triplet GGG or CCC, and so on. We assumed that longer G-tracks should favour G4 folding, whereas longer C-tracks should hinder it. cG and cC scores were the respective sums of all values attributed to Gs and Cs for a given sequence. Thus, the longer the consecutive nucleotide tracks, the higher their score value. For example, a series of three consecutive Gs will have a cG score of 100 [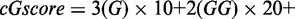 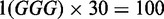], while a series of two consecutive Gs will have a score of 40 [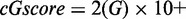 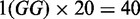]. The cG and cC scores of all study candidates, in both the short and long contexts, were determined (Table 1). As expected, analysis showed that both scores were higher for the longer sequences. Next, to define a parameter integrating both components, the cG score was divided by the cC score, providing the cG/cC score (see Table 1 and Figure 5C). The cG/cC score clustered the G4 folding and non-folding RNA species regardless of transcript length. *P*-values of 0.0105 and 0.0016 were estimated for the short and the long transcripts, respectively. For short transcripts, the *P*-value (0.0105) is slightly lower than that obtained based on the Mfe (0.0106). Our cG/cC score is a novel predictor of RNA G4 folding, which appears to significantly discriminate between folding and non-folding PG4s among a set of different RNA molecules.

### Challenging the cG/cC score

**Figure 6.**
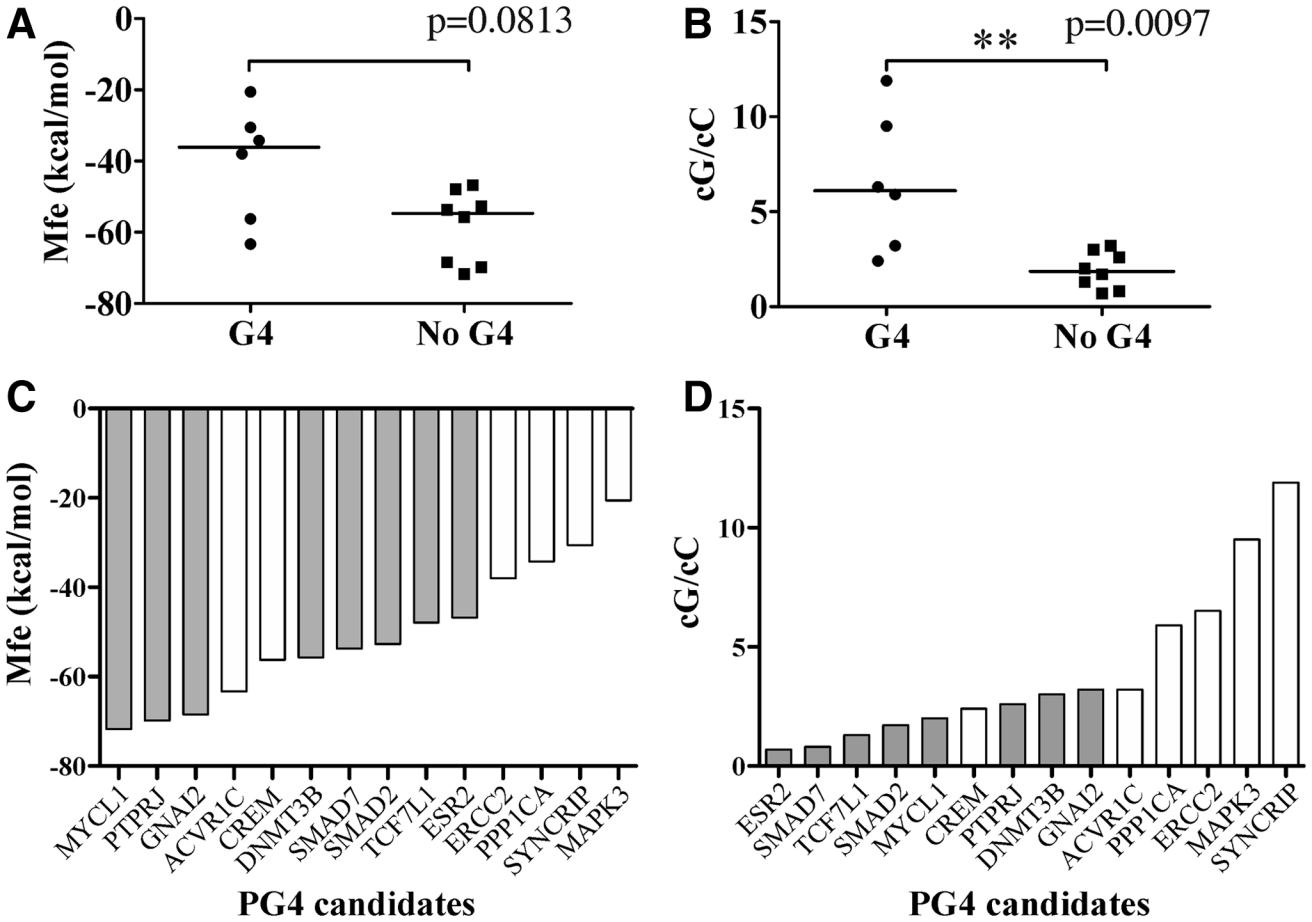
Challenge of predictive values for a new set of 14 PG4s with various genomic contexts. (**A**) Mfe, kcal/mol; and (**B**) calculated cG/cC score. Horizontal lines represent the median for each group. *P*-values were determined with a Mann–Whitney test. **P < 0.01. (**C** and **D**) Bar graphs representing the predicted Mfe value (C) and the cG/cC score (D) of PG4 candidates placed in increasing order. White bars represent folding candidates, whereas gray bars represent non-folding candidates.

To assess the predictive potential of the cG/cC score with respect to G4 folding, 14 novel PG4 candidates retrieved in human 5′-UTRs were selected for analysis. The candidates were chosen for the diversity of their genomic contexts, as illustrated by their predicted Mfe values ranging from −71.7 to −20.6 kcal/mol (Table 2). Both the WT and G/A-mutant versions of each PG4, flanked by ∼50 nt on each side, were synthesized by run-off transcription, 5′-radiolabelled and then submitted to the in-line probing procedure described previously in the presence of 100 mM of either LiCl or KCl. The sequences of each PG4 are given in Supplementary Table S2, and the resulting bar graphs from the in-line probing experiments are presented in Supplementary Figures S13–S26. Only six transcripts supported G4 folding in the presence of K^+^ according to the in-line probing data (Table 2). The other eight transcripts are considered false-positive predictions of the standard sequence algorithm. A bar graph displays the candidates in order of increasing predicted Mfe values, in Figure 6C. As expected, the four candidates with the highest predicted Mfes, i.e. those corresponding to relatively less stable structures, supported G4 folding, whereas the three with the lowest Mfes did not permit G4 folding. However, the seven other candidates with middle Mfe values provided somewhat unexpected results. Mfes for the cluster of G4 folding G4 candidates versus that for the non-folding candidates provide a *P*-value of only 0.0813 (Figure 6A), showing no significant difference between both groups. This is further evidence that Mfe is not an accurate predictive parameter of G4 folding for RNA molecules bearing flanking sequences that mimic the arrangement in naturally occurring transcripts. No total loop length difference was observed between folding and non-folding candidates, indicating that this parameter is also ineffective predictor of G4 folding (data not shown).

**Table 2.** Characteristics of PG4 candidates selected to challenge the predictive parameters

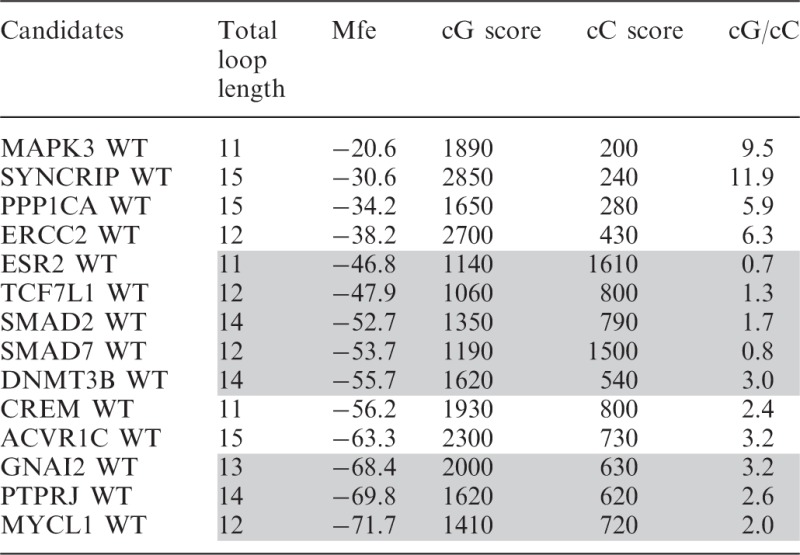

Shaded entries indicate those candidates that cannot fold into G4.

Next, the cG/cC scores were determined for all 14 candidates. Candidates were classified accordingly, the higher the cG/cC score, the more likely G4 folding and vice versa. For 13 out of 14 candidates, the scoring system was a strongly accurate predictor of G4 folding as illustrated in Figure 6D. The single exception, the CREM PG4, displayed an intermediate cG/cC score of 2.4. All other candidates with a high cG/cC score supported G4 folding, whereas low scorers were non-folding. When representing the cG/cC scores of folding and non-folding clusters of PG4 candidates, a *P*-value of 0.0097 indicated discrimination between both groups (Figure 6B). Results thus confirmed the predictive potential of the cG/cC score with respect to G4 folding of RNA transcripts in their genomic contexts. Moreover, the cG/cC score also seemed to limit the number of false-positive predictions. Candidates with the lowest cG/cC scores can readily be regarded as non-folding PG4s.

### Comparison of the cG/cC score with existing parameters and predictive tools

**Figure 7.**
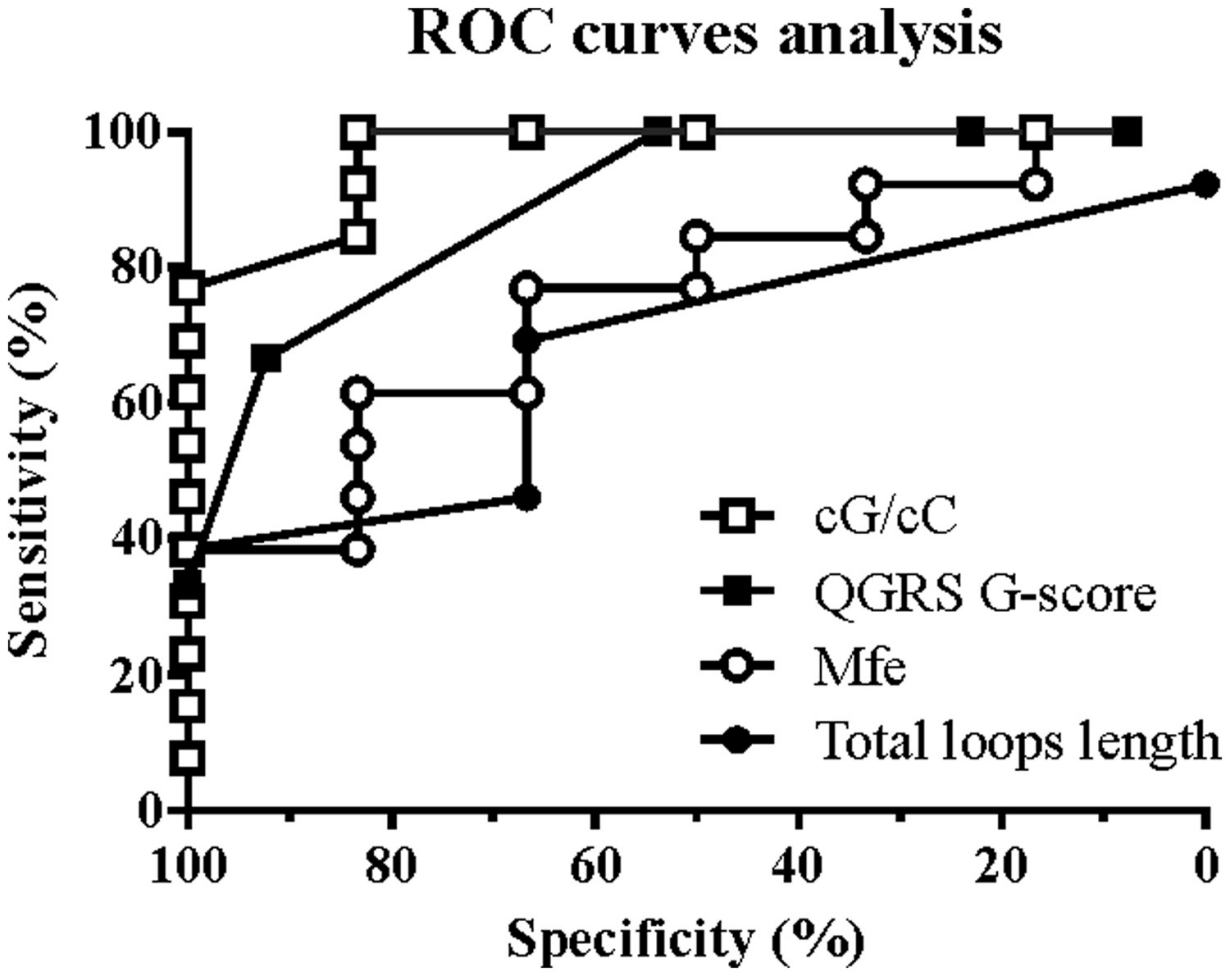
ROC curves analysis of the different predictive parameters for PG4s in their long context. QGRS G-score was evaluated with the QGRS software (29). The AUC is the ability to discriminate between folding and non-folding PG4s. An AUC of 0.5 is a random discriminating value, whereas an AUC of 1 stands for perfect discrimination. The cG/cC score displays the highest AUC and, thus, the highest sensitivity and specificity. ROC curves analyses were performed using GraphPad Prism version 5.02 for Windows, GraphPad Software, San Diego, CA, USA.

This study unambiguously demonstrated that, in contrast to the identification of the RNA PG4s, the accurate prediction of G4 folding requires careful consideration of both upstream and downstream sequences beyond just a few nucleotides on either side of the PG4. More or less distant C-tracks have been suggested to impair G4 folding. While both a conventional secondary structure prediction algorithm and the resulting Mfe parameter appear to be of limited use in determining whether a PG4 located in a relatively long RNA species will fold into a G4, the proposed cG/cC score appears to be a useful alternative. The ratio of cGs to cCs appears to be a good predictor of G4 folding for RNA transcripts. A relatively common way to assess the accuracy of a predictive test and to set a threshold between two conditions (folding and non-folding in the case at hand) is to draw ROC curves. In this kind of analysis, sensitivity, i.e. the fraction of G4 folding candidates with cG/cC scores above the threshold, is plotted against specificity, i.e. the fraction of non-folding candidates with scores below the threshold. The quality of the predictive value is the AUC. An area of 0.5 represents a random, i.e. non-discriminating value, while an AUC of 1 represents a perfect prediction, i.e. generating no false positives or negatives. ROC curves analyses demonstrated that the cG/cC score is both the most sensitive and specific predictor of G4 folding for long RNA transcripts. The cG/cC score also displays higher AUCs compared with total loop length and predicted Mfe (Figure 7, and the AUCs presented in Supplementary Table S5). Use of a scoring system to predict G4 folding has already been proposed by others. Previously, Kikin and collaborators developed the so-called G-score, which was used in combination with the standard sequence algorithm in their QGRS Mapper tool to predict G4 folding (29). The G-score takes into account the number of Gs in G-tracks and the number and arrangement of loop nucleotides. PG4s with longer G-tracks and shorter loop lengths with evenly distributed nucleotides have higher G-scores and are more likely to fold into G4s. However, when applied to the set of RNA PG4 candidates used here, the G-score was less sensitive and specific than the cG/cC score (Figure 7). Since the G-score is mostly based on loop length, this could explain its poorer predictive value compared with the cG/cC score. Despite what was expected from the conclusions of previous studies (10,25), the total loop length was not a significant predictor of G4 folding for the RNA transcripts used in this study. Thus, contrary to their DNA counterparts, RNA PG4 stability and folding potential seem to be relatively less sensitive to loop length and arrangement. However, the neighbouring genomic context and possible competing Watson–Crick structures seem to bear relatively more importance for predicting the folding potential of RNA PG4s. Its single-stranded nature affords RNA PG4s great plasticity, enabling the same molecule to rapidly adopt a multitude of stable secondary structures, whereas in DNA PG4s most of the neighbouring genomic context is constrained by a complementary strand (5). Mfe values obtained with the RNAfold software, which were used as an indication of the relative competitiveness of the neighbouring genomic context, were not efficient predictors of G4 folding for long transcripts. Moreover, it is important to note that this software cannot predict G4 folding. Recently, Lorenz and coworkers published new RNA folding algorithms taking G4s into consideration (30). Thus, we compared this new RNA folding algorithm’s predictions to the cG/cC score predictions, for all of the candidates probed *in vitro* in this study. Most folding predictions were in agreement with our *in vitro* probing results. However, for the 14 candidates used in the cG/cC score challenge (Table 2), four that did not fold into G4s *in vitro* were nevertheless predicted to do so by the new RNA folding algorithm, representing as many false positives. The cG/cC score analysis of the first set of 12 long-transcript candidates (Table 1) permitted to evaluate the predictive ability of different threshold values in terms of sensitivity and specificity (Supplementary Table S6). A threshold of 2.05 had 100% sensitivity and 83.3% specificity. Candidates with a cG/cC score >2.05 are predicted to fold into G4s, whereas candidates with smaller scores are predicted to adopt canonical Watson–Crick structures. Using this cG/cC threshold on the second set of 14 PG4 candidates listed in Table 2 (cG/cC scores were not used to establish the threshold), only three false positives were obtained, i.e. a few less than with the new RNA folding algorithm. To increase specificity of the cG/cC score, a higher threshold value of 3.05 was selected. This new threshold yielded a total of only two discrepancies, i.e. one false positive and one false negative. Taken together, these results show that the new RNA folding algorithm is a fairly effective predictor of G4 folding, but that the cG/cC score can improve and refine predictions.

Future work should focus on assessing G4 folding of a larger set of RNA PG4s within their neighbouring genomic context to further refine the predictive power of cG/cC thresholds. Here, we considered RNA PG4 candidates with genomic context sizes of 15 and 50 nt. Investigation of larger genomic context sizes should help optimize predictive value. Even though not tested per se within the scope of this study, a determinable size limit must apply. Indeed, considering too much genomic context will only dilute the informative value of relative C and G contents. C and G contents nevertheless impact predictive power more strongly than does the exact context size. Therefore, considering a genomic context of 50 nt seems adequate, and fine-tuning of the context size still worthy of future investigations. Because of the intrinsic environmental differences between RNA and DNA PG4s, it is likely that their respective cG/cC thresholds are expected to differ. Window length, or the distance of genomic context considered as impacting G4 folding, is also expected to differ, i.e. be smaller for DNA PG4s because of its double-strandedness. The cG/cC threshold of a given PG4 may also vary owing to its relative position within the genome. For example, PG4s located in coding versus non-coding regions. It is also not impossible that PG4s located in an otherwise unfavourable genomic context or position still form G4s *in vivo* when folding cofactors such as either proteins or small RNAs, bind to neighbouring inhibitory C-tracks as previously proposed [(6); S. Rouleau, JD Beaudoin and JP Perreault, unpublished data]. RNA G4 folding is decidedly more complex than meets the eye. For instance, the same transcript could perhaps generate alternative folding and non-folding isoforms, in differing proportions depending on prevailing cellular conditions. Still another RNA PG4 candidate predicted as non-folding could still yield a small proportion of folding transcripts exerting biological effects despite predictions to the contrary. While the current state of knowledge makes such conjectures premature, future investigations will no doubt continue to shed fascinating insights into the nuances of G4 folding and the prediction thereof. With increasing evidence of ‘atypical’ G4 folding, turning to a predictive scoring system based on guanine density, instead of a standard algorithm, is gaining support and appears to be a suitable avenue to increase the accuracy of G4 folding predictions [(12,31–33); J.L.Mergny, unpublished data]. Prediction of RNA G4 folding based on energy-based models such as the algorithm proposed by Lorenz and coworkers is still in its infancy (30). Currently, relatively little knowledge exists about the energies driving G4 folding compared with canonical Watson–Crick structures. The cG/cC score developed here provides a convenient complementary tool to currently available G4 prediction softwares. Thus the cG/cC score can help discard incorrect folding predictions while essential and more accurate energy-based models are refined.

### Concluding remarks

In summary, the PG4 genomic context, and especially consecutive cytosine content, appears to be one of the main criteria governing G4 folding of RNA molecules. The cG/cC score presented here is representative of this context and based on relative consecutive G to C contents. This helpful new parameter predicts RNA G4 folding with relatively high sensitivity and specificity. It is most useful when used in combination with current G4 prediction tools. The accurate prediction of RNA G4 folding is an essential step towards a better understanding of both their functional roles and biological importance. All of this is part of a much broader challenge aiming to uncover and comprehend the intricate regulatory mechanisms of RNA G4 folding underlying the biological processes of disease and health.

## SUPPLEMENTARY DATA

Supplementary Data available at NAR Online.

## FUNDINGS

Canadian Institute of Health Research (CIHR) [MOP-44022 to J.-P.P.]; Université de Sherbrooke [to the RNA group]. Funding for open access charge: Canadian Institute of Health Research (CIHR) [MOP-44022].

*Conflict of interest statement*. None declared.

## Supplementary Material

Supplementary Data
