# Supplementary material for: New scoring system to identify RNA G-quadruplex folding
Source: Nucleic Acids Res. 2013 Oct 8;42(2):1209–23. doi: 10.1093/nar/gkt904 (PMC3902908; doi:10.1093/nar/gkt904)
Supplement: Supplementary Data [file supp_42_2_1209__index.html]

New scoring system to identify RNA G-quadruplex folding — New scoring system to identify RNA G-quadruplex folding — Supplementary Data 

# New scoring system to identify RNA G-quadruplex folding

## Supplementary Data

files

**Files in this Data Supplement:**

- Supplementary Data - xlsx file
- Supplementary Data - xlsx file
- Supplementary Data - xlsx file
- Supplementary Data - xlsx file
- Supplementary Data - docx file
