# Supplementary material for: New scoring system to identify RNA G-quadruplex folding
Source: Nucleic Acids Res. 2013 Oct 8;42(2):1209–23. doi: 10.1093/nar/gkt904 (PMC3902908; doi:10.1093/nar/gkt904)
Supplement: Supplementary Data [file supp_gkt904_nar-01656-f-2013-File013.docx]

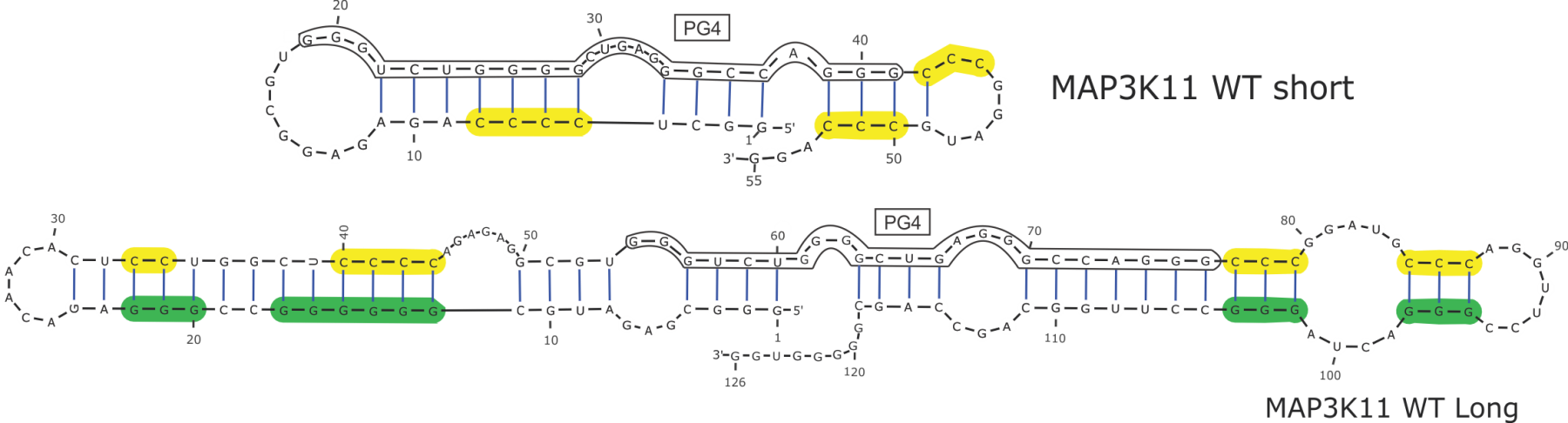


**SUPPLEMENTARY FIGURE S27**

**Beaudoin J-D., Jodoin R., Perreault J-P.**

**Supplementary table S5**. Area under the curve of the different predictive parameters

|  | **Total loop length** | **Mfe** | **cG/cC** | **QGRS G-score** |
| --- | --- | --- | --- | --- |
| **Area under the curve (AUC)** | 0,6795 | 0,7564 | 0,9679 | 0,8974 |

**Supplementary table S6.** Table of sensitivity and specificity percentages for the different cG/cC score thresholds.

| **Threshold** | **Sensitivity (%)** | **Specificity (%)** | **False positives (FP)** | **False negatives (FN)** | **True positives (TP)** | **True negatives (TN)** |
| --- | --- | --- | --- | --- | --- | --- |
| > 0.70 | 100 | 16,67 | 7 | 0 | 6 | 1 |
| > 1.00 | 100 | 50 | 6 | 0 | 6 | 2 |
| > 1.60 | 100 | 66,67 | 5 | 0 | 6 | 3 |
| > 2.05 | 100 | 83,33 | 3 | 0 | 6 | 5 |
| > 2.15 | 92,31 | 83,33 | 3 | 0 | 6 | 5 |
| > 2.40 | 84,62 | 83,33 | 3 | 1 | 5 | 5 |
| > 2.70 | 76,92 | 100 | 2 | 1 | 5 | 6 |
| > 3.05 | 69,23 | 100 | 1 | 1 | 5 | 7 |
| > 3.35 | 61,54 | 100 | 0 | 2 | 4 | 8 |
| > 3.70 | 53,85 | 100 | 0 | 2 | 4 | 8 |
| > 4.25 | 46,15 | 100 | 0 | 2 | 4 | 8 |
| > 4.70 | 38,46 | 100 | 0 | 2 | 4 | 8 |
| > 5.10 | 30,77 | 100 | 0 | 2 | 4 | 8 |
| > 6.00 | 23,08 | 100 | 0 | 3 | 3 | 8 |
| > 7.15 | 15,38 | 100 | 0 | 4 | 2 | 8 |
| > 9.20 | 7,69 | 100 | 0 | 4 | 2 | 8 |

**SUPPLEMENTARY FIGURE LEGENDS**

**Supplementary figures S1 to S26.** In-line probing results of all of the PG4 candidates are shown as bar graphs of the K^+^/Li^+^ ratios. The full length sequence of each candidate is also shown. The PG4 regions are written in blue. Lowercase red guanosines are those mutated to adenines in the G/A-mutants. The lowercase green cytosines are those mutated to adenines in the C/A-mutants. For the TTYH1 PG4 candidate, the lowercase yellow cytosines are those mutated to adenines in the second C/A-mutant version. The guanines boxed in red are those involved in G4 formation.

**Supplementary figure S27**. RNAfold predicted secondary structures for both the MAP3K11 short and long transcripts. The PG4 region is boxed. Inhibitory tracks of cytosines are shown in yellow, and enhancing tracks of guanines are shown in green. In the short transcript the C-tracks are predicted to base-pair with the G-tracks of the PG4, thereby inhibiting its formation. In the longer transcript, the supplementary G-tracks base-pair with the inhibitory C-tracks allowing the G4 to form.
